# New perspectives on integrin-dependent adhesions

**DOI:** 10.1016/j.ceb.2019.12.008

**Published:** 2020-04

**Authors:** Magdalene Michael, Maddy Parsons

**Affiliations:** Randall Centre for Cell and Molecular Biophysics, King's College London, New Hunts House, Guys Cam, London, SE1 1UL, UK

**Keywords:** Integrins, Adhesion, Cytoskeleton, Talin, Kindlin, Adhesion dynamics, Migration, Microscopy

## Abstract

Integrins are heterodimeric transmembrane receptors that connect the extracellular matrix environment to the actin cytoskeleton via adaptor molecules through assembly of a range of adhesion structures. Recent advances in biochemical, imaging and biophysical methods have enabled a deeper understanding of integrin signalling and their associated regulatory processes. The identification of the consensus integrin-based ‘adhesomes’ within the last 5 years has defined common core components of adhesion complexes and associated partners. These approaches have also uncovered unexpected adhesion protein behaviour and molecules recruited to adhesion sites that have expanded our understanding of the molecular and physical control of integrin signalling.

## Introduction

Integrins are a family of 24 heterodimeric receptors that mediate interactions between all cell types and the extracellular matrix (ECM). The formation of integrin-based adhesions has been studied for more than 3 decades, and extensive research has identified the key adaptor proteins and kinases that assemble upon integrin activation to mediate integrin-associated complex (IAC) formation. However, until recently our understanding of the hierarchy of adhesion protein assembly has remained limited. Biochemical approaches have now defined a consensus ‘adhesome’ within adherent cells [1] and high-resolution microscopy has aided in defining the nanoscale assembly of different integrin-containing adhesions [2]. The development of new force- and conformation-sensing biosensors has also provided means to visualise mechanosensing by IAC components and the roles that both internal and external forces play in controlling this. In this review, we highlight the recent developments in understanding mechanisms controlling integrin activation, dynamics and adaptor protein binding in different contexts.

## New perspectives on integrin activation

**Figure 1:**
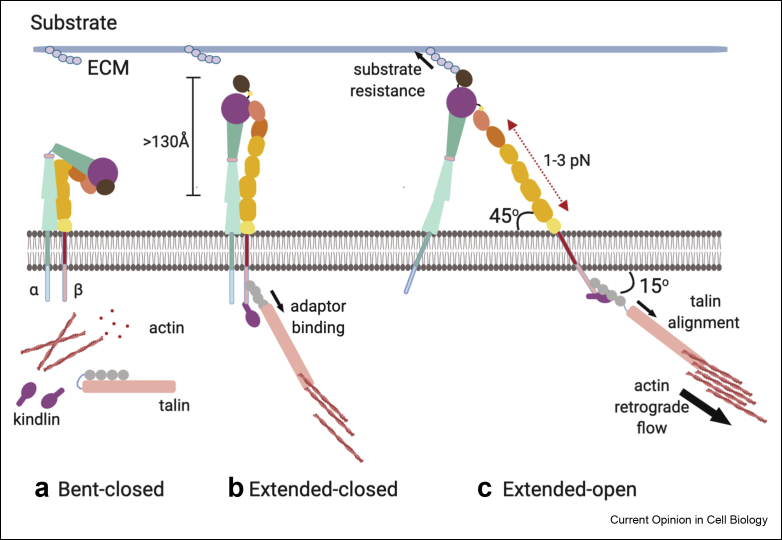
**New insights into integrin activation.** Integrins exist in three states: **(a)** bent-closed, an inactive conformation where the integrin is not engaged with its ECM ligand; **(b)** ‘extended-closed’, a low affinity, intermediate state that may arise from talin and/or kindlin binding; **(c)** ‘extended-open’, elicited by simultaneous binding of ECM ligand and intracellular adaptors associated with the actin cytoskeleton. Intracellular adaptor binding leads to a >130 Å extension of integrin conformation [4,12]. Resistive forces from ligand binding and cytoskeletal adaptor interactions (thin black arrows) exert 1–3 pN tensile forces on the integrin (red double arrows). The direction of actin retrograde flow (thick black arrows) generates tension on talin positioning it 15^0^ to the plasma membrane and drives the tilting of the integrin β subunit to an angle ∼45^0^ to the plasma membrane aligning it with the F-actin filaments [19,30]. This extended, tilted integrin orientation establishes equilibria along its force-bearing axis and stabilises the high-affinity ligand binding state. Based predominantly on data taken from LFA-1 and ICAM-1 binding studies. ICAM-1, Intercellular Adhesion Molecule-1.

Extensive work, largely performed *in vitro*, has provided a framework that integrins are positioned orthogonal to the cell membrane and exist in multiple conformations: bent-closed (inactive), extended-closed (active, low affinity) and extended-open (active, high affinity) conformations. The extended, open conformation has been the focus of most studies and thought to be required only for ligand binding and adhesion. However, single particle cryoelectron microscopy has now identified a role for the extended-closed conformation of αvβ8 and αvβ3 integrins, stabilised by a structural change in the α subunit, in ligand surveillance [3]. A study using interference photoactivation localisation microscopy to determine conformational changes of LFA-1 (αLβ2) during intercellular adhesion molecule-1 (ICAM1) binding further showed tilting occurs within the heterodimer in the extended-open conformation [4], contrary to conventional models (Figure 1). Combined mathematical modelling and molecular dynamics simulation of integrin conformational changes have also enabled the exploration of the effects of long- and short–range interactions on full length integrin extension to better understand the structural transitions adopted by integrins during various modes of activation [5]. Moreover, evidence is emerging that different integrin heterodimers in the same cell can show distinct conformations and ligand binding kinetics. Biophysical analysis suggests that α4β1 is more highly tuned to activation at lower force or adaptor concentrations than α5β1, potentially reflecting the ability of α4β1 to mediate transient adhesion of leukocytes [6]. Furthermore, α4β1 and α5β1 show significantly greater reliance on cytoplasmic-induced conformational changes for their ligand binding affinities compared with αVβ6 [7]. Supporting the emerging notion of differential integrin-specific modes, Litvinov et al., [8] have also shown that specific regions within the transmembrane domain of β3 integrin can dictate their α integrin pairing (either with αv or αIIb) and consequently its ligand binding specificity in platelets, leading to distinct physiological outcomes. These new lines of evidence suggest integrin conformational changes are more diverse than previously thought, offering potential means for more rapid cellular response to specific ligands.

**Figure 2:**
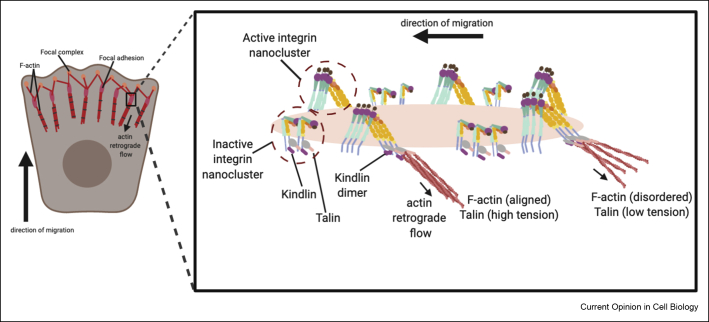
**Molecular architecture of integrin within focal adhesions.** Integrins within focal adhesions organise into nanoclusters segregated into active and inactive states [17,20]. Active integrin clusters adopt a tightly ordered distribution aligning with the F-actin retrograde flow [19] (black arrows), indicative of a stable, ECM-bound population; clusters of inactive integrin are less organised and dispersed, characteristic of a freely diffusing, mobile pool. Despite differences in spatial organisation, both integrin cluster types associate with talin and kindlin. These discrete integrin clusters may reflect the tension anisotropy observed within focal adhesions. As kindlin-2 dimers have been implicated in integrin activation [10] and talin can exists in varying tensional states within focal adhesions [29], we could assume that clusters of active integrin would contain kindlin dimers with talin under high tension, whereas inactive clusters would contain monomeric kindlin with talin under low tension. Organised parallel bundles of F-actin at membrane proximal regions of the focal adhesion correlates with high talin tension, whereas lower talin tension is observed when F-actin bundles are less aligned [29].

Inside-out signalling, whereby intracellular signals promote integrin ligand binding conformations, is mediated by talin and kindlin association to the proximal and distal regions of β cytoplasmic tail. This leads to integrin activation, clustering and recruitment of other intercellular adaptor proteins promoting adhesion strengthening; however the precise mechanisms involved still remain unclear [9]. Detailed structural analysis has provided new insight into mechanisms underpinning adaptor protein binding to control integrin activation. Recent studies have uncovered a second TTV/STF (amino acid) sequence binding site on β integrins that allows for simultaneous binding of a kindlin-2 F2 dimer, which is required for integrin activation [10] (Figure 2). Molecular dynamics simulations further suggest that forces applied to integrins, strengthen association between the kindlin dimers and integrin cytoplasmic tails through a catch-bond mechanism, similar to that described for talin [11], however details of how and where kindlin-2 forms a dimer remain to be determined.

Integrin activation is a finely tuned process as indicated by the level of open-active integrins on the surface of resting immune cells (0.1–0.9%) [12]. Indeed, talin-mediated integrin activation examined using fluorescence polarisation on integrin domain fragments have revealed that binding of talin in the absence of force, gives graded regulation of integrin activation, even in the allosterically stabilised high-affinity extended-open state. By contrast, application of cytoskeletal force via adaptor proteins or ligand binding stabilises integrin extension to enable ultrasensitive activation [6]. These studies therefore offer potential new ways to consider integrin adaptor protein binding kinetics during activation initiation and adhesion maturation.

Contextual regulation of integrin adaptors such as talin is also an emerging important consideration in the understanding of integrin inside-out signalling. *In vivo* analysis demonstrates that talin engages both integrin-binding sites and lies parallel to the epithelial membrane in the *Drosophila* wing, but lies orthogonal to the membrane in muscle, using only the four-point-one, ezrin, radixin, moesin (FERM) domain integrin-binding site, potentially because of higher mechanical forces exerted at the latter [13]. Recruitment of talin to the membrane has been previously thought to require a Rap1–RIAM cascade, but recent evidence suggests that where RIAM levels are limiting, Rap1 can bind directly to talin at the plasma membrane to relieve autoinhibition both *in vitro* and *in vivo* [14,15]. Conversely, SH3 and multiple ankyrin repeats (SHANK) proteins that act as negative regulators of integrin activation have been shown to bind and sequester Rap1 to limit talin-mediated integrin activation [16]. Thus, context-specific signals can control the balance of local integrin activators/inhibitors to position talin as the primary activation trigger, followed by recruitment of kindlin for further strengthening upon force application.

## Discrete integrin signalling domains

The emergence of super-resolution microscopy techniques has revealed that IACs are not homogeneous assemblies as previously thought but are instead heterogenous macromolecular complexes with discrete arrangements of active and inactive integrins. Single-molecule microscopy and photo-activated localization microscopy (PALM) have demonstrated that IACs comprise substructures (0.01–0.1 μm^2^) containing <100 molecules [17], and similar high-density discrete β1 integrin-containing structures have been identified using scanning electron-assisted dielectric-impedance microscopy [18]. Ligand-engaged integrins (αvβ3 and β1) have also been visualised as tightly spaced nanoclusters within IACs, aligned along the focal adhesion long axis, an organisation dependent upon F-actin retrograde flow via talin binding [19,20] (Figure 2). These highly ordered substructures of active integrin reflect a more stable ECM-engaged pool with potentially enhanced sensitivity to cellular forces compared with the disorganised nonaligned clusters of inactive integrin. Interestingly, while these two subsets of integrins exist as discrete clusters, they both contain talin, vinculin and kindlin-2 [20], raising further questions about how this partitioning occurs (Figure 2).

In addition to showing distinct substructures, super-long single-molecule tracking has also revealed that integrins experience ‘temporary arrest of lateral diffusion’ at IACs, a process requiring traction forces generated through ECM linkages and actomyosin activity [24]. Growing focal adhesions exhibit longer temporary arrest of lateral diffusions at distinct sites, corresponding to regions of highest traction, further supporting the notion of distinct subdomains within IACs. Kank proteins have also been recently identified as regulators of discrete adhesion subdomains [21]. Identified through proteomic screens, Kank binds the talin rod domain specifically within the lateral border of focal adhesions at sliding adhesions beneath the nucleus. The talin–Kank complex reduces talin–actin association, thereby reducing forces across integrins and ligand binding, leading to adhesion slippage and attenuation of migration [22,23]. Adhesions are therefore not homogeneous assemblies as initially assumed but rather contain distinct regions of specific protein complex hubs that dictate integrin stability and may play a role in tuning subcellular responses to different mechanochemical environments.

## Mechanosensing and force generation by integrins

Integrins are continuously experiencing forces from both sides of the plasma membrane and although known to be key integrators of mechanical signals, the precise way in which forces couple integrins to cell signalling machinery remained unclear [24,25]. Recent evidence suggests that intracellular tensile forces and ligand binding can lead to integrin activation that is ultrasensitive to lower levels of forces compared with cytoskeletal adaptor binding alone [12]. Notably, β3 integrins show longer, force-dependent residence times in IACs in response to tension, whereas β1 integrins maintain uniform times [26]. These differences similarly act to fine tune rigidity sensing as each integrin can activate distinct downstream pathways. Mechanical coupling and force transmission of talin to integrin and actin is crucial for adhesion stability and downstream signalling [27]. Talin is also critical for adhesion reinforcement and refines subcellular responses by restricting mechanical activation and creating signalling anisotropy required for cell polarity [28]. Correlative imaging approaches have revealed gradients of tension across talin within growing adhesions, with highest levels of tension being seen closest to theplasma membrane [29] (Figure 2). Combined fluorescence resonance energy transfer (FRET) and correlative electron tomography also showed regions of high F-actin alignment corresponding to these regions of high talin tension, indicating interdependence between actomyosin-mediated force generation and IAC formation (Figure 2). Integrins subjected to forces coalign with F-actin retrograde flow and orient at the plasma membrane with a tilt angle of ∼45^0^ [30] (Figure 1). Interestingly, traction forces exerted by individual integrin receptors also align with cytoskeletal adaptors at 45^0^ with respect to the substrate plane [31], further confirming a co-ordinated relationship between integrin activation, force and IAC alignment (Figure 1).

The molecular clutch model is widely recognised as a mechanism for integrin engagement. Modelling has confirmed that during maximum spreading, which occurs at intermediate viscosity on soft substrates, integrin engagement and clutch reinforcement occur, and substrate relaxation is on a timescale between clutch binding and IAC lifetime [32]. On stiffer substrates, clutch loading is saturated, and viscosity exerts no effect. A biphasic model of integrin adhesion to substrates has also been determined using AFM analysis of α5β1 integrins, whereby initial rapid strengthening of adhesions is followed by a slower binding phase once mechanical load threshold is achieved, analogous to a catch bond [33]. These findings suggest a model where talin-mediated linkage to F-actin is required for the response to mechanical load, with kindlin subsequently strengthens IACs by stabilising the active integrin conformation.

Integrin mechanosensing is also emerging as a key regulator of physiological processes. For example, increased mechanical stiffness of the mesoderm triggers collective neural crest migration in the developing *Xenopus* embryo, a response requiring the integrin–vinculin––talin complex [34]. IACs also regulate apical forces in the *Drosophila* amniosera by counteracting apical membrane tension to achieve a balance of cell–cell and cell–ECM adhesions required for dorsal closure [35]. Force sensing via integrins also plays a key role in the vasculature, where compressive forces exerted by red blood cells on platelets provide a mechanical cue to activate integrins, enhancing the αIIbβ3 integrin-fibrinogen on-rate leading and platelet adhesion via increased Ca^2+^ and PI-3kinase signalling [36]. Similarly, the mechanical stretching of the endothelia during vascular perfusion can activate β1 integrins, driving angiocrine signals for hepatocyte survival, liver growth and regeneration [37]. The mechanical environment of the cell, both internal and external, is arguably, therefore, equally important as the ligand availability/type in dictating cycles of integrin activation.

## New roles for integrins

Integrins are well characterised IAC components, but recent evidence suggests they may also exist in clusters distinct from classical focal adhesions. An example of this is seen in αvβ5 containing ‘reticular’ adhesions that are long-lived, integrin-based structures lacking both talin and F-actin [38]. These adhesions have a distinct molecular profile from other adhesion types and comprise of endocytic and membrane regulatory proteins that associate with retraction fibres in a PI(4,5)P2-dependent manner. Notably, reticular adhesions are preserved during all stages of mitosis suggesting a co-ordinated link between these adhesions, cell division and postmitotic spreading. Tension gauge tethers have also identified uniformly distributed integrins outside focal adhesions that can contribute to bulk cellular forces despite lower reliance on F-actin and microtubule activities [39], again suggesting that widely studied classical IACs may not be the only sites for integrin-dependent signalling.

Aside from commonly studied adhesion-dependent signals, forces on integrins are emerging as key controllers of metabolic pathways to modulate energy production [40]. Adenosine monophosphate-activated protein kinase (AMPK), a major regulator of metabolism, has been shown to positively regulate integrin-mediated actin protrusion at the migrating edge of cells where increased mitochondrial activity is required [41]. Conversely, AMPK can inhibit integrin activation through control of tensin expression leading to increased fibrillar adhesion formation [42]. Although these findings may appear contradictory, it is likely that the role for AMPK in integrin regulation is context dependent, supporting the notion that metabolic-sensing by AMPK can locally control specific integrin-binding partners to elicit migratory responses to changing environmental conditions.

In addition to operating within IACs, integrins can also co-operate with signalling at cell––cell junctions. ZO-1 within tight junctions has been shown to enhance α5β1 binding to fibronectin at the free edge of cell monolayers, which decreases resistance to external forces [43]. Similarly, E-cadherin–mediated force loading initiates an epidermal growth factor receptor (EGFR)-PI-3kinase–driven signalling cascade that activates α5β1 at the base of the cell, enabling Abl kinases to recruit vinculin to sites of high tension to create a positive feedback loop that reinforces cell stiffening [44]. There is also increasing evidence to suggest that integrins may signal directly from cell–cell junctions. In *Drosophila* egg chamber follicle cells, Rab10-mediated collagen IV secretion occurs initially at lateral membranes and is subsequently deposited at the basement membrane during migration, leading to uniform ECM distribution [45]. Collagen IV accumulation has also been observed at cell–cell adhesions in *Drosophila* adipocytes, along with integrins, talin, PINCH and integrin-linked kinase (ILK), and interestingly, integrin overexpression has been shown to increase collagen at intercellular contacts, thereby reinforcing these adhesions [46]. Thus, emerging evidence of high ECM levels between adjacent cells certainly suggests integrins may play, yet, unknown roles within lateral adhesion sites that will be important to explore in future.

## Concluding remarks and future challenges

Adoption of new methods has provided unprecedented new insight into integrin activation and adhesion dynamics. The rapid acceleration in the development of new microscopy-based approaches and biosensors to study protein dynamics, positioning and interactions at the nanoscale will provide means to facilitate new discoveries in defining protein function simultaneous with nanoscale positioning and associated effects on cell behaviour. However, although the key players in adhesions are now documented, significant gaps remain in our understanding of multiprotein complex formation and dynamics and how both internal and external forces act to spatiotemporally refine these interactions. Moreover, as integrin signalling in cells within 3D environments differs from those on 2D surfaces, the extension of biochemical and imaging approaches to more physiological settings represents a future challenge to the field if we are to understand integrin behaviour *in vivo* and potentially target integrins for therapeutic benefit in pathological settings [47].

## Author contributions

MM and MP both wrote the original draft; MM drafted the figures with initial guidance from MP. Both authors read and approved final version.

## Conflict of interest statement

Nothing declared.
